# Prevalence of stroke and stroke related risk factors: a population based cross sectional survey in southwestern China

**DOI:** 10.1186/s12883-019-1592-z

**Published:** 2020-01-07

**Authors:** Xingyang Yi, Hua Luo, Ju Zhou, Ming Yu, Xiaorong Chen, Lili Tan, Wei Wei, Jie Li

**Affiliations:** 1 Department of Neurology, The People’s Hospital of Deyang City, No 173, North Taishan Road, Deyang, 618000 Sichuan China; 2 Department of Neurology, The Affiliated Hospital of Southwest Medical University, Luzhou, 646000 Sichuan China; 3 Department of Neurology, the Suining Central Hospital, Suining, 629000 Sichuan China; 4 Centre of rehabilitation, the People’s Hospital of Deyang City, Deyang, 618000 Sichuan China

**Keywords:** Stroke, Epidemiology, Risk factors, Health care

## Abstract

**Background:**

Stroke and its risk factors epidemiological survey can help identify individuals at higher risk and therefore promote stroke prevention strategies. The aim of this study was to estimate the current prevalence of stroke and high risk stroke population, and evaluate stroke associated risk factors in southwestern China.

**Methods:**

This was a multi-center, cross sectional survey in southwestern China from May 2015 to September 2015. The eight communities were selected at random, and 17,413 residents aged ≥40 years volunteered to participate in this survey. Data were collected through face-to-face survey using a structured questionnaire. Five hundred twenty-one participants with incomplete questionnaires on stroke history or risk factors records were excluded.

**Results:**

A total of 16,892 people included in analysis. The overall prevalence of stroke was 3.1% (95% CI 2.6–3.9%), 17.1% of participants were the high risk stroke population. After full adjustments, hypertension, diabetes, dyslipidemia, overweight, lack of exercise and family history of stroke were significantly associated with overall stroke and ischemic stroke. The largest contributor was hypertension (population-attributable risk 23.6%), followed by dyslipidemia, physical inactivity, family history of stroke, diabetes, and overweight. However, only hypertension (OR = 3.66, 95% CI 1.82–8.23) was significantly associated with hemorrhagic stroke.

**Conclusions:**

The prevalence of stroke and high risk stroke population was high among adults aged ≥40 years in southwestern China. Hypertension, dyslipidemia and lack of exercise were stronger contributors for stroke, these findings suggest that individual-level and population-level interventions for these leading risk factors are necessary to prevent stroke.

## Background

Stroke is a leading cause of adult mortality and disability, and there are approximately 3 million new stroke cases every year in China [1, 2]. In the past several decades, the incidence of stroke has decreased because of effective strategies for preventing cerebrovascular risk factor and good health services in developed countries. However, the converse has been revealed for developing countries. In recent years, the economic climate in China has changed considerably, the epidemiologic features of stroke in China have likely changed substantially in the last decades [3]. However, rare comprehensive community-based surveys have been completed since the 1990s to support these changes in China [4], except several hospital-based registration studies [5].

The China National Stroke Screening Survey (CNSSS) is one community-based stroke surveillance program in China [6]. The aims of the survey are to monitor stroke trends, identify high risk factors for stroke, investigate the current epidemiologic features of stroke, and assess intervention policies in China. The results of CNSSS in part showed that the adjusted stroke prevalence was 2.06% in adults aged ≥40 years, the incidence of stroke in China increased rapidly in 2002 to 2013 in China [1]. Given that China had the highest number of prevalent cases of stroke in the world [7], and more recently reported by results of the Global Burden of Disease Study [8], more vigorous and effective interventions are needed to prevent stroke.

In China, the prevalence of stroke is different in different regions and between rural and urban areas [9]. The prevalence of stroke in rural areas sharply increased between 2003 and 2013, whereas in urban areas the prevalence was stable in the same period. A north-to-south geographical gradient in stroke prevalence, incidence and mortality is apparent, with numbers being lowest in the south and highest in the northeast of China [10]. A recent study also showed that stroke prevalence exhibited a noticeable north-south gradient (1097.1, 917.7, and 619.4 in the north, middle, and the south, respectively), and stroke prevalence was higher in the rural regions than in the urban (945.4 versus 797.5) regions in China [11]. However, with regarding to mortality-to-incidence ratio (MIR) of stroke, the MIR is the highest in the southwest and the lowest along the eastern and southern coasts [10]. These regional differences in MIR indicate striking disparities in both access to and quality of stroke care across the country [12].

Accurate provincial and regional-level stroke prevalence estimates are very important for research planning and targeted strategies for stroke prevention and management. Sichuan is located in Southwestern of China, and is an economically underdeveloped area, with an area of 486,000 km^2^ and 73.02 million inhabitants. The prevalence and incidence of stroke in Sichuan province was very high in China according to CNSSS [13], and since then, rare studies have revisited this important public health issue. In recent years, the economic climate, people’s dietary habits, awareness and health concepts in Sichuan have changed considerably. Considering these developments, the epidemiology of stroke in Sichuan may have changed. Thus, we hypothesized that the prevalence of stroke in Sichuan could increase over time, the features of different population groups could vary, and certain risk factors, including metabolic diseases and unhealthy lifestyle, would have major contributions to burden of stroke. Hence, we performed one community-based stroke survey in 8 communities in Sichuan province according to CNSSS program between September 2014 and September 2015. This study aims to estimate the stroke prevalence and the pattern of its related risk factors, and fill the information gap in this field in Sichuan.

## Methods

### Study design and participants

This population based cross sectional study was part of the CNSSS (grant No. 2011BAI08B01) and was carried out in the Sichuan province from May 2015 to September 2015. All methods of survey were performed in accordance with the CNSSS program and approved by the Stroke Screening and Prevention Programme of the National Health and Family Planning Commission of China. The survey protocol was reviewed and approved by the Ethics Committee of the participating hospitals (the People’s Hospital of Deyang City, the Affiliated Hospital of Southwest Medical University, and Suining Central Hospital), and informed consent was obtained from all participants during recruitment.

A cluster survey method was used, and 8 communities in Sichuan were selected at random. The stroke surveillance methods were compiled by the National Center for Stroke Control and Prevention. More details on the organization and implementation can be found at the official website [13]. Briefly, the CNSSS is a cross-sectional survey with a 2-stage stratified sampling framework. We only screened all residents for ages ≥40 years in each community, because the prevalence of stroke is very low among younger adults [14]. All participants were people who had lived in the county for at least 6 months, and were initially screened using a structured face-to-face questionnaire by interviewers. The questionnaire included demographic characteristics (eg, age, gender, education level and employment), stroke related behavioural factors (eg, drinking, smoking, exercise habits and diet), personal and family medical history of stroke and chronic diseases (ie, hypertension, diabetes mellitus, dyslipidemia and atrial fibrillation [AF]), and physical examination (eg, height, weight, resting blood pressure). More detailed information regarding the lifestyle, related diseases, and laboratory examinations (such as fasting blood glucose [FBG], lipid, electrocardiogram [ECG], and carotid ultrasonography) was also obtained from the individuals who had experienced stroke and from the participants who were identified to be at a high risk for stroke.

### Definitions of stroke and evaluation of risk factors

According to the World Health Organization criteria, stroke was defined as “rapidly developing clinical signs of focal (or global) disturbance of cerebral function, lasting more than 24 h or leading to death, with no apparent cause other than that of vascular origin [15]”. In this survey, stroke history and stroke types were established by a combination of self-reporting and the judgment of a physician or neurologist according to neuroimaging (including brain computed tomography scan and magnetic resonance imaging). Subtypes of stroke included ischemic stroke and hemorrhagic stroke. By definition, patients with a history of transient ischemic attack only were excluded.

According to CNSSS program [1, 6], the eight conventional stroke risk factors were assessed in the CNSSS questionnaire included behavioral factors (overweight/obesity, smoking, physical inactivity), family history of stroke, and biomedical factors (hypertension, diabetes, dyslipidemia, and AF). Eight stroke related risk factors were defined as follows: hypertension was defined as a self-reported history or the use of antihypertensive drugs, or the average of two resting systolic blood pressure readings of ≥140 mmHg and/or diastolic blood pressure ≥ 90 mmHg in the field survey [16]. Diabetes mellitus was defined as the use of insulin and/or oral hypoglycaemic medications, or a self-reported history of diabetes or FBG ≥7.0 mmol/L in the field survey [17]. Dyslipidemia was defined as using a lipid-lowering medication or having one or more of the following in the field survey: triglycerides (TG) ≥ 1.70 mmol/L, cholesterol (TC) ≥ 5.18 mmol/L, and low-density lipoprotein cholesterol (LDL-C) ≥ 3.37 mmol/L [18]. AF was defined as reported by the respondent or diagnosed by ECG in the field survey. Current smoking (≥1 cigarette per day) was defined by subjects’ self-report. Body mass index (BMI) was calculated as weight (kg) divided by height squared (m^2^), and overweight or obesity was defined as BMI ≥26 kg/m^2^ [19]. Physical inactivity was defined as physical exercise < 3 times a week for < 30 min each time, and this included industrial and agricultural labour [20]. A family history of stroke was restricted to immediate family members.

Subjects with at least three of the aforementioned eight stroke related risk factors or a history of stroke were classified as the high risk population for stroke. The risk assessment scales for stroke referred were designed by the CNSSS, and have been proved to have good reliability and validity compared with the modified scale of the Framingham Stroke Profile (FSP), and can be used as an evaluation tool for stroke risk assessment [21].

### Data cleaning procedures and quality control

**Fig. 1:**
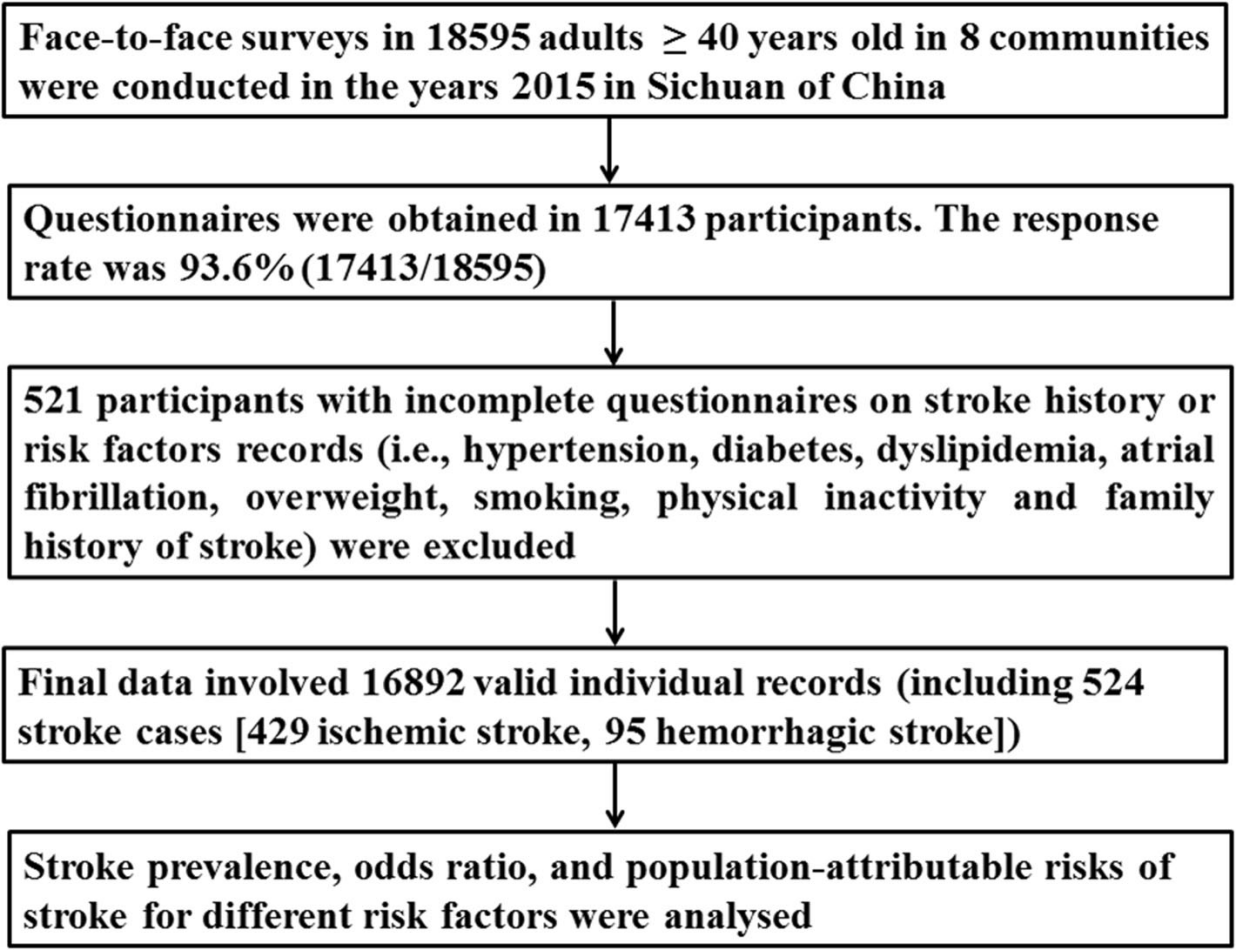
Data preparing and cleaning process in this survey

The detailed data cleaning procedure and quality control according to the CNSSS is presented in Fig. 1. Briefly, 18,595 participants volunteered to participate in the face-to-face survey, questionnaires were obtained in 17,413 participants. The response rate was 93.6% (17,413/18595). Five hundred twenty-one participants with incomplete questionnaires on stroke history or risk factors records were exclude. Finally, 16,892 valid individual records (including 524 stroke cases [429 ischemic stroke, 95 hemorrhagic stroke]) were enrolled. After the data cleaning procedure, there were no missing values in the variables assessed.

The interviewers were physicians or neurologists from community hospitals, who had at least 5 years of education in medicine. The quality of the measurements and data collection were maintained by implementing uniform training and standardized protocols. The staff involved in the survey were trained by the CNSSS program and passed the examination at the end of train. All data were entered electronically into a data terminal that was directly connected with the CNSSS database.

### Sample size estimates and statistical analysis

According to the CNSSS, screening should cover at least 1% of the local residents aged ≥40 years. There were 167,553 residents aged ≥40 years in the 8 communities according to the sixth national population census in 2010 [22], 10% of the targeted population, therefore, the expected sample size was 16,755. The sample size (N) necessary for this cross sectional study was calculated based on a prevalence (p) of stroke of 2.37% among adults aged ≥40 years in China [6], with a 0.5% uncertainty level (d), using the formula n = tα^2^pq/d^2^ (t = 1.96, α = 95% for both sides; q = 1- p), we calculated a required sample size of 16,765. Considering a lost to follow-up rate of 10%, the planned sample size was 18,628 (16,765/0.90). Finally, 18,595 participants aged ≥40 years participated in this survey.

Descriptive analyses were conducted to determine the distribution of the demographic data and risk factors in the study population using SPSS 17.0 (SPSS Inc. New York, New York, USA). Categorical variables are presented as proportions and were compared using χ2 tests between different subgroups. The adjusted odds ratios (ORs) and 95% confidence intervals (CIs) of each risk factor for stroke prevalence rate were derived using unconditional multivariate logistic regression models, fully adjusting for all other potential confounders, including age, sex, education, urban/rural residency, smoking, physical inactivity, overweight, hypertension, diabetes, dyslipidemia, AF, and family history of stroke.

We calculated population-attributable risks (PARs) of stroke, ischemic stroke and hemorrhagic stroke from the model using the Bruzzi method for determining the confounder-adjusted PAR [23], which has been applied in many previous studies. The 95% CIs were evaluated for the PARs according to the previously described procedure [24].

All tests were two-sided, and *P* value < 0.05 was considered statistically significant.

## Results

The baseline characteristics of the study population were shown in Table 1. In total of 16,892 participants, there were 524 stroke cases (3.1%), 2893 participants (17.1%) were the high risk stroke population. Of the 524 stroke cases, ischemic stroke accounted for 429 (81.9%), hemorrhagic stroke accounted for 95 (18.1%). The overall prevalence of stroke was 3.1% (95% CI 2.6–3.9%).

**Table 1:** Demographic characteristics of study populations and stratified prevalence of stroke [n(%)]

Variables	High risk population for stroke	Overall stroke	Ischemic stroke	Hemorrhagic stroke
Total (*n* = 16,892)	2893 (17.1%)	524 (3.1%)	429 (2.5%)	95 (0.6%)
Sex				
Male(*n* = 5411)	1370 (25.3%)	201 (3.7%)	158 (2.9%)	43 (0.8%)
Female(*n* = 11,481)	1523 (13.3%)	323 (2.8%)	271 (2.4%)	52 (0.5%)
*P* value	< 0.001	0.003 (9.94)	0.029 (4.65)	0.006 (7.68)
Age, y				
40–49(*n* = 3524)	318 (9.0%)	37 (1.0%)	30 (0.8%)	7 (0.2%)
50–59(*n* = 5106)	686 (13.4%)	103 (2.0%)	87 (1.7%)	16 (0.3%)
60–69(*n* = 5803)	1125 (19.4%)	228 (3.9%)	188 (3.2%)	40 (0.7%)
70–79(*n* = 2183)	629 (28.8%)	131 (6.0%)	106 (4.9%)	25 (1.1%)
≥ 80(*n* = 276)	135 (48.9%)	25 (9.1%)	18 (6.5%)	7 (2.5%)
*P* value	< 0.001	< 0.001	< 0.001	< 0.001
Residence				
Urban(*n* = 8889)	1361 (15.3%)	260 (2.9%)	203 (2.3%)	57 (0.6%)
Rural(*n* = 8003)	1532 (19.1%)	264 (3.3%)	226 (2.8%)	38 (0.5%)
*P* value	< 0.001	0.186 (1.96)	0.026 (4.96)	0.173 (2.09)
Education				
Primary school or below (*n* = 8331)	1948 (23.4%)	369 (4.4%)	310 (3.7%)	59 (0.7%)
Junior middle school(*n* = 3312)	568 (17.1%)	106 (3.2%)	83 (2.5%)	23 (0.7%)
Senior middle school (*n* = 3111)	228 (7.3%)	33 (1.1%)	23 (0.7%)	10 (0.3%)
College or above (*n* = 2138)	149 (0.7%)	16 (0.7%)	13 (0.6%)	3 (0.1%)
*P* value	< 0.001	< 0.001	< 0.001	0.017 (10.6)
Overweight/obesity				
Yes(*n* = 8615)	1545 (17.9%)	355 (4.1%)	290 (3.4%)	65 (0.8%)
No(*n* = 8277)	1348 (16.3%)	169 (2.0%)	139 (1.7%)	30 (0.4%)
*P* value	0.004 (8.07)	< 0.001	< 0.001	< 0.001
Smoking				
Yes(*n* = 3676)	748 (20.3%)	139 (3.8%)	114 (3.1%)	25 (0.7%)
No(*n* = 13,216)	2145 (16.2%)	385 (2.9%)	315 (2.4%)	70 (0.5%)
*P* value	< 0.001	0.006 (7.2)	0.017 (5.98)	0.283 (1.16)
Physical inactivity				
Yes(*n* = 8226)	1752 (21.3%)	305 (3.7%)	251 (3.1%)	54 (0.7%)
No(*n* = 8666)	1141 (13.2%)	219 (2.5%)	178 (2.1%)	41 (0.5%)
*P* value	< 0.001	< 0.001	< 0.001	0.124 (2.54)
Hypertension				
Yes(*n* = 7018)	2082 (29.7%)	313 (4.5%)	246 (3.5%)	67 (1.0%)
No(*n* = 9874)	811 (8.2%)	211 (2.1%)	183 (1.9%)	28 (0.3%)
*P* value	< 0.001	< 0.001	< 0.001	< 0.001
Diabetes				
Yes(*n* = 2754)	780 (28.3%)	106 (3.8%)	94 (3.4%)	12 (0.4%)
No(*n* = 14,138)	2113 (14.9%)	418 (3.0%)	335 (2.4%)	83 (0.6%)
*P* value	< 0.001	0.013 (6.1)	0.003 (10.1)	0.323 (0.94)
Dyslipidemia				
Yes(*n* = 3235)	822 (25.4%)	141 (4.4%)	108 (3.3%)	33 (1.0%)
No(n = 13,657)	2071 (15.2%)	383 (2.8%)	321 (2.4%)	62 (0.5%)
*P* value	< 0.001	< 0.001	< 0.001	< 0.001
Atrial fibrillation				
Yes(*n* = 232)	72 (31.0%)	14 (6.0%)	13 (5.6%)	1 (0.4%)
No(n = 16,660)	2821 (17.1%)	510 (3.1%)	416 (2.5%)	94 (0.6%)
*P* value	< 0.001	0.009 (6.7)	0.006 (8.9)	0.138 (2.2)
Family history				
Yes(*n* = 2224)	528 (23.7%)	86 (3.9%)	55 (2.5%)	31 (1.4%)
No(n = 14,668)	2365 (16.1%)	438 (2.9%)	374 (2.5%)	64 (0.4%)
*P* value	< 0.001	0.027 (4.98)	0.984 (0.05)	< 0.001

The prevalence rates stratified by demographic characteristics and risk factors are crude estimates

The prevalence rate (unadjusted [crude]) of stroke was significantly higher in men than in women (3.7% vs 2.8%, *P* = 0.003) and in individuals with a primary school level of education or below than in individuals with a college level of education or above (4.4% vs 0.7%, *P* <  0.001). The prevalence rate of stroke increased with age (*P* <  0.001). However, there was no statistically significant difference in stroke prevalence rate between rural populations and urban populations (3.3% vs 2.9%, *P* = 0.186). Similar to the overall prevalence rate of stroke, the prevalence rate of ischemic stroke and hemorrhagic stroke was also significantly higher in men than in women (2.9% vs 2.4%, *P* = 0.029, and 0.8% vs 0.5%, *P* = 0.006, respectively), and the prevalence rate of ischemic stroke and hemorrhagic stroke also increased with age (all *P* <  0.001 for ischemic stroke and hemorrhagic stroke), but decreased with educational level (*P* <  0.001 and *P* = 0.017, respectively). In addition, the prevalence rate of ischemic stroke was higher in rural populations than in urban populations (2.8% vs 2.3%, *P* = 0.026), but there was no statistically significant difference in hemorrhagic stroke rate between rural and urban residents (0.5% vs 0.6%, *P* = 0.173) (Table 1).

The crude prevalence rates of stroke were significantly different according to all risk factors, including hypertension, diabetes, dyslipidemia, AF, overweight, smoking, physical inactivity and family history of stroke. Stratified by risk factors, the crude stroke prevalence rate was the highest in the residents with AF (6.0%), followed by those with hypertension (4.5%), dyslipidemia (4.4%), overweight (4.1%), family history of stroke (3.9%), diabetes and smoking (3.8%), and physical inactivity (3.7%) (Table 1).

Adjusted ORs for the individual risk factors by multivariable logistic regression model were showed in Table 2. The multivariable logistic regression showed that multiple characteristics were significantly associated with stroke, including age, gender, hypertension, diabetes, dyslipidemia, overweight, lack of exercise and family history of stroke. The strongest risk factors for overall stroke were hypertension (OR = 3.38, 95% CI 1.65–5.23), dyslipidemia (OR = 2.15, 95% CI 1.39–3.17) and physical inactivity (OR = 1.95, 95% CI 1.37–3.37), followed by family history of stroke (OR = 1.87, 95% CI 1.32–2.33), diabetes(OR = 1.57, 95% CI 1.33–2.14), and overweight (OR = 1.36, 95% CI 1.13–1.69). These patterns were consistent for ischemic stroke (Table 3). However, the multivariate analyses model found that only hypertension (OR = 3.66, 95% CI 1.82–8.23) was significantly associated with hemorrhagic stroke (Table 4).

**Table 2:** Odds ratios and population-attributable risk factors for overall stroke by multivariable regression models

Variables	Reference groups	Control groups	Odds ratio (95% CI)	*P* value	PAR (%) (95% CI)
Sex	Female	Male	1.42 (1.31–2.02)	0.004	NA
Age, y	40–49	50–59	1.86 (1.42–2.86)	< 0.001	NA
		60–69	4.07 (2.48–4.77)		NA
		70–79	5.36 (2.66–6.89)		NA
		≥80	6.21 (2.94–7.35)		NA
Residence	Rural	Urban	0.92 (0.83–1.02)	0.324	NA
Education	Primary school or below	Junior middle school	1.02 (0.86–2.03)	0.436	NA
		Senior middle school	0.98 (0.87–1.53)		NA
		College or above	0.89 (0.84–1.12)		NA
Overweight/obesity	No	Yes	1.36 (1.13–1.69)	0.015	6.3 (5.33–8.54)
Smoking	No	Yes	1.32 (0.67–1.87)	0.652	5.2 (0.92–5.83)
Physical inactivity	No	Yes	1.95 (1.37–3.37)	< 0.001	10.3 (8.3–12.6)
Hypertension	No	Yes	3.38 (1.65–5.23)	< 0.001	23.6 (19.8–28.7)
Diabetes	No	Yes	1.57 (1.33–2.14)	0.023	6.9 (5.92–7.33)
Dyslipidemia	No	Yes	2.15 (1.39–3.17)	< 0.001	10.8 (8.8–10.23)
Atrial fibrillation	No	Yes	1.33 (0.76–2.52)	0.433	2.2 (0.95–3.82)
Family history	No	Yes	1.87 (1.32–2.33)	0.008	8.2 (9.13–16.8)

*CI* confidence interval, *PAR* population-attributable risk, *NA* not applicable

**Table 3:** Odds ratios and population-attributable risk factors for ischemic stroke by multivariable regression models

Variables	Reference groups	Control groups	Odds ratio (95% CI)	*P* value	PAR (%) (95% CI)
Sex	Female	Male	1.13 (1.02–1.56)	0.013	NA
Age, y	40–49	50–59	1.67 (1.22–2.13)	< 0.001	NA
		60–69	3.12 (1.92–3.25)		NA
		70–79	4.36 (2.44–5.38)		NA
		≥80	5.54 (2.38–6.49)		NA
Residence	Rural	Urban	0.96 (0.87–0.97)	0.512	NA
Education	Primary school or below	Junior middle school	0.94 (0.82–1.43)	0.262	NA
		Senior middle school	0.92 (0.84–1.25)		NA
		College or above	0.90 (0.86–1.04)		NA
Overweight/obesity	No	Yes	1.34 (1.08–1.72)	0.035	6.8 (5.62–8.45)
Smoking	No	Yes	1.18 (1.34–1.64)	0.267	3.1 (0.97–3.31)
Physical inactivity	No	Yes	1.97 (1.33–3.02)	0.008	9.7 (9.82–15.93)
Hypertension	No	Yes	3.45 (1.77–7.56)	< 0.001	20.2 (16.5–24.4)
Diabetes	No	Yes	1.65 (1.31–2.35)	0.026	5.7 (5.22–7.54)
Dyslipidemia	No	Yes	2.03 (1.42–3.96)	< 0.001	11.2 (7.84–9.93)
Atrial fibrillation	No	Yes	1.28 (0.97–2.25)	0.089	2.4 (1.01–5.62)
Family history	No	Yes	1.94 (1.26–3.02)	0.011	7.9 (8.32–13.64)

*CI* confidence interval, *PAR* population-attributable risk, *NA* not applicable

**Table 4:** Odds ratios and population-attributable risk factors for hemorrhagic stroke by multivariable regression models

Variables	Reference groups	Control groups	Odds ratio (95% CI)	*P* value	PAR (%) (95% CI)
Sex	Female	Male	0.91 (0.86–1.46)	0.364	NA
Age, y	40–49	50–59	1.62 (0.75–2.64)	0.143	NA
		60–69	2.13 (1.02–4.68)		NA
		70–79	1.96 (0.92–3.12)		NA
		≥80	1.73 (0.96–2.97)		NA
Residence	Rural	Urban	0.98 (0.68–1.35)	0.462	NA
Education	Primary school or below	Junior middle school	1.12 (0.76–2.25)	0.275	NA
		Senior middle school	0.93 (0.85–1.58)		NA
		College or above	0.92 (0.77–1.62)		NA
Overweight/obesity	No	Yes	1.11 (0.67–1.65)	0.215	2.6 (0.99–3.84)
Smoking	No	Yes	1.23 (0.96–1.96)	0.464	1.5 (0.89–1.93)
Physical inactivity	No	Yes	1.09 (0.89–2.15)	0.233	1.9 (0.93–2.65)
Hypertension	No	Yes	3.66 (1.82–8.23)	< 0.001	15.3 (13.2–17.5)
Diabetes	No	Yes	1.26 (0.96–2.56)	0.435	2.5 (0.96–3.45)
Dyslipidemia	No	Yes	1.28 (0.86–1.87)	0.289	2.3 (0.97–2.81)
Atrial fibrillation	No	Yes	1.29 (0.91–1.59)	0.364	2.1 (0.98–2.62)
Family history	No	Yes	1.63 (0.99–3.04)	0.086	5.8 (1.02–8.32)

*CI* confidence interval, *PAR* population-attributable risk, *NA* not applicable

Hypertension (23.6%), dyslipidemia (10.8%), and physical inactivity (10.3%) were the 3 risk factors with the largest contributions to the PAR of stroke after full adjustments (Table 2). Family history of stroke, overweight/obesity, diabetes, smoking, and atrial fibrillation each accounted for < 10% of the total PAR of stroke. Furthermore, the results of PAR for ischemic and hemorrhagic stroke were shown in Table 3 and Table 4.

## Discussion

In this study, the results showed that high risk populations for stroke were very common in Sichuan of southwestern China. We have also identified a high prevalence of stroke and stoke related risk factors among adults aged ≥40 years, and found that age, male, diabetes, hypertension, dyslipidemia, lack of exercise, overweight were associated with a high prevalence of stroke and ischemic stroke. These findings were consistent with those of previous studies on stroke prevalence in China [1, 20].

The prevalence of stroke in Sichuan was higher than the nationwide findings [1, 6], but was lower than in northeast China [20]. The prevalence of stroke increased with age, and this was consistent with other studies [25]. In the past decades, rapid economic development in China has increased life expectancies, the percentage of elderly people in population has also increased. The effect of population ageing on the stroke prevalence and disability-adjusted life years (DALYs) has become more and more serious in China. Our results also revealed that the prevalence of stroke was higher in men than in women, this was in accordance with other previous studies in China [25, 26]. Stroke prevalence was higher in the rural regions than in the urban in China [1, 9, 11]. Income, the proportions and amount of awareness for stroke, and treatment and control of hypertension were lower in rural residents than urban residents, which may substantially affect the stroke prevalence [11]. In addition, smoking prevalence and salt intake is higher among rural residents than urban residents, and personal daily consumption of vegetables and fruit is lower in rural areas than urban areas [9, 11]. These may explain the differing stroke prevalence between urban and rural areas in China. However, in contrast with previous studies [1, 9, 11], there was no significant difference in stroke prevalence between urban and rural areas in this survey. This indicated that disparities in residence was consistent with rapid economic development, and economic gap is narrowing between urban and rural areas.

In this survey, we found that hypertension, dyslipidemia and physical inactivity were strongest risk factors for overall stroke, with PAR of 23.6, 10.8 and 10.3%, respectively, and followed by family history of stroke, diabetes, and overweight. Hypertension appeared to be the most important contributor for stroke, however, only 29.7% (2082/7018) of patients with hypertension receiving antihypertensive treatment in this survey. Our finding was consistent with previous studies [27, 28]. In China, the prevalence of hypertension substantially increased from 1979. However, the proportions of awareness, treatment, and control of hypertension decreased or remained constant in China from 2000 to 2010 [1, 9], whereas they have substantially increased in the high-income countries [29], these may affect the prevalence of stroke. Our results showed that the prevalence of dyslipidemia and the proportions of physical inactivity were higher than the national levels in recent studies [1, 30, 31]. The prevalence of hyperlipidemia increased from 8% in 1985 to 11.2% in 2014 in China [1, 32]. Population in physical activity decreased by 25% from 1991 to 2011 [33], and increased energy intake of fat may increase in obesity and dyslipidemia in the Chinese population [34], consequently increasing the risk of overweight or obese and the related metabolic abnormalities. The high prevalence of hypertension and dyslipidemia might be ascribed to dietary preferences and low physical activity in residents of Sichuan Province. In contrast to previous studies [27, 28], smoking only accounted for 5.2% of the PAR for stroke in this study, this may be due to the significant decrease in the prevalence of smoking from 30.4% in 1980 to 24.2% in 2012 [35]. In addition, according to other studies, the prevalence of smoking was higher in male than in female [1, 36]. In Sichuan, many male residents leave their home town to external work, this may be one of reasons of low the proportions of men and low the prevalence of smoking in this survey. Based on our aforementioned findings, clinical control of hypertension and hyperlipidemia, and behavioral interventions for metabolic and lifestyle risk factors are very important for preventing stroke.

Compared with previous Chinese studies [4], prevalence of hemorrhagic stroke was comparatively lower, and only hypertension was significantly associated with hemorrhagic stroke in this survey. The reasons for this difference may due to differences in study design, and the high case-fatality of hemorrhagic stroke could partially explain our observation of fewer hemorrhagic stroke patients in survivors [14]. However, because the DALYs for hemorrhagic stroke were very higher than those for ischemic stroke in China, the burden of hemorrhagic stroke remains, despite its low prevalence [7, 27, 36]. This finding also indicates that antihypertensive therapy is very important to decrease prevalence of hemorrhagic stroke.

Although this was the most recent investigation of stroke, high risk population for stroke, and associated risk factors in Sichuan, this cross sectional survey involved a large representative sample of the Sichuan. Several limitations of this study should be noted. First, this study was the cross sectional study, and there may have the recall bias because of the self-reported questionnaire. Second, we only screened residents for ages ≥40 years; therefore, our present results cannot be generalized to all population groups in Southwestern China. Third, as known to all, atrial fibrillation is an important risk factor for stroke, it increases stroke risk by 5 times [37]. The prevalence of atrial fibrillation was very low in the survey, the respondents’ atrial fibrillation status was based on self-report and ordinary ECG, which may have underestimated paroxysmal atrial fibrillation [38]. Finally, some other risk factors (such as alcohol intake, air pollutants and dietary patterns,) are shown to contribute to stroke risk [36], we were unable to involve them in present analyses due to lack of these information in this survey. Furthermore, our study did not prospective follow-up of participants and collect information about mortality or cause of death, and assess intracranial arterial disease in participants. In future studies, we plan to prospective follow-up participants, collect information about mortality or cause of death, and investigate stroke incidence in participants.

## Conclusion

In this study, we have identified a high prevalence of stroke and high risk population for stroke, and related risk factors of stroke among adults aged ≥40 years in southwestern China. Hypertension, dyslipidemia and lack of exercise were stronger contributors for overall stroke, followed by family history of stroke, diabetes, and overweight, and these factors are appropriate targets for the primary prevention of stroke. Individual-level and population-level interventions to control these leading risk factors of stroke identified in present study are needed to reduce the burden of stroke in southwestern China.

## Data Availability

The datasets used and/or analysed during the current study are available from the corresponding author on reasonable request.

## Abbreviations

AF: atrial fibrillation
BMI: body mass index
CI: confidence interval
CNSSS: china national stroke screening survey
DALYs: disability-adjusted life years
ECG: electrocardiogram
FBG: fasting blood glucose
LDL-C: low-density lipoprotein cholesterol
MIR: mortality-to-incidence ratio
OR: odds ratio
PARs: population-attributable risks
TC: cholesterol
TG: triglycerides
